# Etiological Association Between Psoriasis and Thyroid Diseases

**DOI:** 10.7759/cureus.12653

**Published:** 2021-01-12

**Authors:** Srilatha Eapi, Rupak Chowdhury, Odunayo S Lawal, Nimisha Mathur, Bilal Haider Malik

**Affiliations:** 1 Internal Medicine, California Institute of Behavioral Neurosciences & Psychology, Fairfield, USA; 2 Pathology, California Institute of Behavioral Neurosciences & Psychology, Fairfield, USA; 3 Pediatrics, California Institute of Behavioral Neurosciences & Psychology, Fairfield, USA

**Keywords:** psoriasis, hypothyroidism, autoimmunity, propylthiouracil, thyroid function tests, psoriatic arthiritis

## Abstract

Psoriasis is a chronic relapsing/remitting autoimmune disease affecting skin and fingernails. It is associated with many other autoimmune diseases such as rheumatoid arthritis, celiac disease, Crohn's disease, and thyroid diseases. Two important autoimmune thyroid diseases - Hashimoto's thyroiditis (hypothyroidism) and Grave's disease (hyperthyroidism) - affect the body's significant organs such as the brain, muscles, digestive function, and the skin. Although some studies have established the connection between psoriasis and thyroid diseases with autoimmunity, our article provides an in-depth analysis of the connection between these two diseases and other common etiological factors associated with them, along with autoimmunity.

We reviewed articles from PubMed using regular keywords and Medical Subject Headings (MeSH) keywords and finalized 45 articles to find an association between these two diseases. These articles showed that this association is more prevalent in obese patients and late-onset psoriasis. Most of the articles showed a positive association, but few articles showed no connection between them. However, there is no concrete explanation to prove the association due to limited research; additional studies are necessary. It requires the attention of both clinicians and researchers to develop a universal drug that will work on both diseases, and also thyroid evaluation could be included in psoriatic patient care so that there is a possibility to decrease cost and efforts while treating these diseases.

## Introduction and background

Psoriasis (PSO) is a chronic, relapsing/remitting autoimmune inflammatory skin disorder. It presents as red, scaly patches, plaques with variable severity affecting elbows, knees, scalp, palms/soles, and nails. Psoriasis affects up to 2-4% of the studied population [1]. A genetic predisposition is significant in the pathogenesis of PSO; however, environmental factors can activate the disease [2].

Psoriasis is associated with a variety of other morbidities, including diabetes mellitus, metabolic syndrome, cardiovascular disease [3], and other inflammatory autoimmune diseases such as psoriatic and rheumatoid arthritis, celiac disease, thyroid disease, and Crohn's disease [4]. Psoriatic arthritis (PsA) is an inflammatory arthritis of the joints in 7-26% of psoriatic patients [5].

Thyroid hormones and abnormalities affect the heart, brain development, digestive system, bones, muscles, skin, and other organs. The prevalence of thyroid dysfunction in the adult population ranges from 1% to 10% and is even higher in selected groups [6].

In this review, we are discussing psoriasis and thyroid diseases and how they are associated with each other because there are only a few studies have examined a link between these two diseases, even though it has been established that some thyroid diseases, like Graves' disease and Hashimoto's thyroiditis, have a causal relationship with autoimmune disease. Also, there is a significantly higher prevalence of thyroid peroxidase antibodies (TPO Ab), thyroglobulin antibodies (Tg Ab), pseudo-nodularity, hypo-echogenicity, and increased vascularity [7] observed in psoriatic patients.

In this article, we found that some studies show a positive correlation between these two diseases, and some studies show there is no association. More prospective and retrospective studies are needed to assess the association between them before any conclusion can be made.

If researchers and physicians can establish a clear association of these diseases, there is a possibility of developing a standard treatment for both diseases. By including thyroid ultrasound and thyroid antibodies in the evaluation of psoriasis, early diagnosis, and treatment of any thyroid abnormalities can be possible.

Our study aims to evaluate the probable common etiological factors associated with psoriasis and thyroid disorders.

## Review

Methods

PubMed is our primary database to collect and review articles. We neither use any inclusion/exclusion criteria nor depend on age, sex, gender, race, and demographics to select the articles. We collected, integrated, reviewed, produced data to find an association between psoriasis and thyroid diseases. A set of regular keywords (Table 1) and Medical Subject Headings (MeSH) keywords (Table 2) are used to collect the data, and the results are mentioned in the tables below.

**Table 1:** Regular keywords

KEYWORDS	DATABASE	NUMBER OF RESULTS
Psoriasis	PubMed	49,146
Hypothyroidism	PubMed	44,229
Autoimmunity	PubMed	45,135
Thyroid Function Tests	PubMed	18,082
Propylthiouracil	PubMed	573
Psoriatic Arthritis	PubMed	9,927

**Table 2:** MeSH keywords

KEYWORDS	DATABASE	NUMBER OF RESULTS
Psoriasis	PubMed	11
Hypothyroidism	PubMed	08
Autoimmunity	PubMed	24

Results

On PubMed, the keyword psoriasis generated 49,146 articles; hypothyroidism resulted in 44,229 articles; autoimmunity gave 45,135 articles, thyroid function tests brought up 18,082 articles, propylthiouracil resulted in 573 articles, and psoriatic arthritis gave 9,927 articles. We searched psoriasis, hypothyroidism, and autoimmunity under Medical Subject Headings (MeSH) keywords criteria, resulting in 11, 8, and 24 articles, respectively.

We finalized and reviewed 45 articles. Among the 45 articles, 39 showed a positive association and only six articles showed no association. In this article, we try to show how thyroid disorders and psoriasis are related to each other in terms of clinical features, pathogenesis, diagnostic tests, and treatment modalities. Five articles showed thyroid hormones' effect on psoriasis pathogenesis, two articles showed genetic association (long non-coding ribonucleic acid (RNA), signal transducer and activator of transcription 4 (STAT4) gene polymorphism play a crucial role in both the diseases), immunological association (interleukin 17 (IL17), C-X-C motif chemokine ligand 10 (CXCL10), CXCL9, C-C motif chemokine ligand 2 (CCL2), and CCL22-associated inflammation are evidenced in both the disorders) is found in six articles, reactive oxygen species-related pathogenesis observed in two articles, and positive thyroid peroxidase antibodies, thyroglobulin antibodies and Hashimoto's thyroiditis ultrasound features in psoriasis patients are exhibited in five articles. We also noticed that propylthiouracil, which has been used as a first-line treatment for hyperthyroidism, clears psoriatic lesions by various mechanisms in six articles, and it didn't induce clinical hypothyroidism or caused any serious adverse effects such as agranulocytosis or acute hepatotoxicity. In two articles, after thyroidectomy, there is marked improvement in psoriatic skin lesions.

During our review process, we found that some articles established a more prevalent association in young females. In contrast, other studies had no gender preference in the prevalence of thyroid antibodies among psoriatic patients. We also found an increase in adipokines-derived cytokines such as leptin in both thyroid disorder and psoriasis; this explains the prevalence of TPO Abs in obese psoriatic patients than non-obese. TPO Abs were also found in late-onset psoriasis than early-onset psoriasis. Some studies showed that psoriasis severity affects thyroid diseases; some studies showed that it did not.

Discussion

*Positive Association*: Many prospective and retrospective studies with a varying number of patients and studies show that there is a significant positive association between psoriasis and Hashimoto's thyroiditis, as evidenced by:

1) Clinical features (cutaneous manifestations): Patients with thyroid diseases such as hypothyroidism are associated with many cutaneous manifestations that draw both endocrinologists' and dermatologists' attention. The association of both psoriasis and thyroid diseases is indicated in a healthy 15-year-old girl with psoriasiform lesions, abscesses, and extremely severe hypothyroidism; all were presented simultaneously. In this patient, there is a significant improvement in the skin lesions upon normalization of thyroxine (T4) levels following initiation of thyroid supplementation, which illustrates a direct association [8].

2) Pathophysiology: There are several pathophysiological mechanisms shared between psoriasis and thyroid diseases.

a) Role of Hormones: The thyroid hormones and their receptors expressed in the skin are essential in stimulating skin proliferation by acting as endogenous inhibitors of skin inflammation [9], most likely due to interference with activator protein 1 (AP-1), nuclear factor kappa-light-chain-enhancer of activated B cells (NF-κB), and signal transducer and activator of transcription 3 (STAT3) activation, and by increasing the level of epidermal growth factor (promotes keratinocyte proliferation and differentiation) [10].

Arguments for the aggravating effect of thyroid hormones in psoriasis are the following [11]: Psoriasis is intensified by the excessive production of thyroid hormones [12], the free thyroxine is increased significantly in psoriatic patients [13], the patients with thyroiditis had more extended disease periods [13], and in severe psoriasis, there are increased levels of thyroid-stimulating hormone (TSH) [10].

As mentioned above, thyroid hormones have a significant effect on psoriasis pathogenesis. It is evidenced by propylthiouracil antiproliferative and immunomodulatory effects on psoriasis and psoriasis resolution after thyroidectomy.

b) Genetic Association: Long non-coding RNA (lncRNA), with a size larger than 200 nucleotides, is a new class of non-coding RNA. Recent evidence has revealed that lncRNAs play a crucial role in the regulation of immunological functions and autoimmunity. Herein, Wu et al., in their review, mentioned the recent findings of lncRNA regulation in immune functions and the development of autoimmunity and autoimmune diseases, including systemic lupus erythematosus (SLE), type 1 diabetes mellitus (T1DM), multiple sclerosis (MS), rheumatoid arthritis (RA), autoimmune thyroid disease (AITD), psoriasis, polymyositis/dermatomyositis (PM/DM) and Crohn's disease (CD) [14].

Liang et al. conducted a metanalysis to investigate whether combined evidence shows the association between STAT4 rs7574865 polymorphism and autoimmune diseases. This meta-analysis demonstrates that the STAT4 rs7574865 T allele confers susceptibility to SLE, RA, T1DM, systemic sclerosis (SSc), juvenile idiopathic arthritis (JIA), primary antiphospholipid syndrome (APS), inflammatory bowel diseases-ulcerative colitis (IBD-UC), autoimmune thyroid diseases, and psoriasis, supporting the hypothesis of an association between STAT4 gene polymorphism and subgroup of autoimmune diseases [15].

However, the association between STAT4 rs7574865 single nucleotide polymorphism and different autoimmune diseases remains controversial and ambiguous. Some studies found that the STAT4 rs7574865 polymorphism increases autoimmune thyroid diseases in the general population but not in psoriatic patients [16].

c) Immunological Association: Several inflammatory pathways have been described in AITD and PSO and PsA, suggesting commonality in pathophysiology. The important cytokine, IL17, stimulates keratinocyte to produce several chemokines resulting in sustained inflammation in the skin, skin barrier disruption, and epidermal hyperproliferation [17]. IL17 is found to play an essential role in autoimmune thyroiditis too. Expression of various other chemokines, such as CXCL10, CXCL9, CCL2, and CCL22, additionally has been observed in both psoriatic disease and AITD [18,19,20]. CCL2 and CCL22 show the transformation from a type 1 T helper (Th1) to type 2 T helper (Th2) immunity, which can be observed in advanced disease stages of both PsA and Graves' disease [18,19,20]. A disarranging in NF-κB signaling pathway, an important feature of many autoimmune diseases, has been illustrated in both AITD and psoriasis [21]. Another potential pathophysiological affiliation is that psoriatic skin lesion has abnormal thyroid and retinoid signaling [22].

d) Reactive Oxygen Species Role: It is well documented that oxidative stress (reactive oxygen species (ROS) generated by the immune system) is one of the significant factors involved in the pathogenesis of psoriasis [23,24]. ROS influences cellular events such as cell proliferation, apoptosis, cell differentiation, and immune response, and these events are altered in psoriasis patients [23,24]. Elevated ROS levels are associated with thyroid dysfunction, especially hyperthyroidism, which enhances the ROS production that perturbs the ROS steady-state environment to facilitate the cellular damage or damage to the cellular components as also reported in psoriasis patients [25]. Therefore, it is assumed that in psoriasis, cells or cellular components are continuously exposed to oxidative stress so that alterations in conformation and function of these cellular components may occur, resulting in modification of their biological properties. Because of these, a study was performed in the College of Medicine, Qassim University, Buraidah, Saudi Arabia between May 2014 and February 2015 to investigate the role of ROS-induced epitopes on albumin and thyroid antigens in psoriasis autoimmunity [26]. This study showed that the anti-ROS-epitopes-HSA-IgGs showed cross-reactions with thyroid antigen, thyroglobulin, and their oxidized forms. The high degree of specific binding by psoriasis IgGs (immunoglobulin G) to ROS-epitopes-HSA, ROS-thyroid antigen, and ROS-thyroglobulin was observed. Finally, it is determined that ROS's structural alterations in albumin and thyroid antigens generate unique neo-epitopes, which could be a factor for the induction of autoantibodies in psoriasis.

3) Antibodies and ultrasound features: There is a higher prevalence of TPO Ab, Tg Ab, and Hashimoto's thyroiditis ultrasound features such as hypoechogenicity, pseudo-nodularity, and increased vascularity in patients with psoriasis. Alidrisi et al. mentioned in their article [7] that both antibodies and ultrasound features were found in psoriasis patients, especially in patients with late-onset psoriasis and/with obesity.

This association is more frequently seen in severe psoriasis, female, and adult patients [10, 27]. Risk factors for the development of thyroid dysfunction are TSH within normal range but at the higher limit, small thyroid volume, and positive TPO Abs, especially in psoriasis women [28,29].

This association between these two diseases draws physicians' attention (Endocrinology and Dermatology) and researchers as it profoundly impacts medical research and patient care in the view that it better to include thyroid ultrasound and thyroid antibody, especially TPO Ab and thyroid function follow-up in the evaluation of a patient with psoriasis so that we can diagnose early and give suitable treatments for thyroid function abnormalities.

4) Drug effects and surgery: Treatment of psoriasis and thyroid diseases affect thyroid hormones and psoriasis severity vice versa.

a) Etanercept: Tumour necrosis factor alpha (TNF-alpha) is a pro-inflammatory cytokine involved in the pathogenesis of inflammatory disorders like psoriasis. Etanercept is a TNF alpha blocker used in the treatment of psoriasis. Roman et al., in their article [30], mentioned that etanercept increases the serum levels of inactive TNF alpha in psoriatic patients with improvement of skin lesions, there is a strong negative correlation between TSH levels and TNF alpha, and the mean level of free thyroxine (FT4) is significantly higher in psoriatic patients without etanercept treatment.

b) Anti-thyroid thioureylenes (propylthiouracil and methimazole): Anti-thyroid thiourylenes, especially propylthiouracil (PTU), have been used as a first-line treatment in hyperthyroidism. Their role in the treatment of psoriasis has been recognized for many years. PTU treatment for six to eight weeks clears psoriatic lesions indicated by decreased Psoriasis Area and Severity Index (PASI) score by several mechanisms [31].

Prolactin has a hyperproliferative effect on keratinocytes, and it is increased in psoriasis. Malligarjunan et al. described the PTU role as an antiproliferative agent in their article [32]. After treatment with PTU for six to 12 weeks, it is observed that psoriatic lesions are cleared, and prolactin is decreased in the respective patients. PTU binds to hepatic T3 receptor, and it is possible that PTU binding to the ligand-binding site which is generally occupied by T3 impairs the transcription by disabling the effect of T3 and by squelching retinoic X receptor heterodimer development with other steroid receptors superfamilies like the peroxisome proliferator-activated receptor, retinoic acid receptor, and vitamin D receptors [33]. Adenosine deaminase (ADA) is a marker for T-cell activation. T-cell activation has been implicated in psoriasis's pathogenesis. After treatment with PTU, elevated baseline ADA activities in skin and plasma seem to be lower, and decreased baseline erythrocyte ADA was higher [34]. PTU also has antioxidant potential along with antiproliferative and immunomodulatory effects indicated by its role in increased lipid peroxidation and decreased antioxidant activity (SOD) in psoriasis [35]. Malondialdehyde (MDA), the end product of lipid peroxidation, superoxide dismutase (SOD) and glutathione peroxidase (GSH-Px), and antioxidant enzymes were measured in plasma, erythrocytes, and skin biopsies of psoriatic patients before and after eight weeks of oral treatment with PTU (300 mg/day) or PTU/thyroxine (25 mg). Increased baseline MDA in all samples was found to be lower. The increased plasma SOD levels in skin and erythrocytes of the study groups were found to be higher and lower respectively in all patients after the treatment. In psoriasis, there is an increased expression of involucrin, a precursor protein that leads to abnormal keratinocyte differentiation, PTU attributable to the downregulation of involucrin in psoriasis [36].

PTU has many side effects, but when used in psoriasis, it did not induce clinical hypothyroidism or caused any serious adverse effects like agranulocytosis or acute hepatotoxicity [37]

Psoriasis is a chronic debilitating disease; many agents used in this disease management are toxic, expensive, and need constant monitoring. There is always a need for another drug that offers less toxicity, more beneficial, and affordable. PTU is found as an alternative drug that shows improvement in psoriatic lesions without significant side effects [Table 3].

**Table 3:** Various studies on propylthiouracil and its various mechanism of actions in the treatment of psoriasis PTU: propylthiouracil; PASI: Psoriasis Area and Severity Index; ADA: adenosine deaminase; MDA: malondialdehyde; SOD: superoxide dismutase; GSH-Px: glutathione peroxidase

Author	Article title (year of publication)	Propylthiouracil and its various mechanism of actions in the treatment of psoriasis
Malligarjunan et al. [32]	Clinical Efficacy of Propylthiouracil and Its Influence on Prolactin in Psoriatic Patients (2011)	PTU treatment for six weeks and 12 weeks cleared psoriatic lesions indicated by decreased PASI score (p < 0.001). Patients before treatment showed significantly increased Prolactin (PRL) levels (male p < 0.01, female p < 0.001) when compared to controls, which was found to decrease significantly (male p < 0.01, female p < 0.001) after 12 weeks.
Elias et al. [33]	Anti-thyroid Thioureylenes in the Treatment of Psoriasis (2004)	Propylthiouracil (PTU) has been shown to bind to the hepatic T3 receptor. PTU (6-n-propyl-2-thiouracil) binding to the ligand-binding site usually occupied by T3 may impair transcription by disabling the effect of T3 and squelching retinoic X receptor heterodimer development with other receptors pertaining to steroid receptor superfamily like the peroxisome proliferator-activated receptor, retinoic acid receptor, and vitamin D receptors.
Kose et al. [34]	Effect of Propylthiouracil on Adenosine Deaminase Activity and Thyroid Function in Patients with Psoriasis (2001)	Adenosine deaminase (ADA) is a marker for T-cell activation, T-cell activation has been implicated in the pathogenesis of psoriasis. After treatment with PTU, elevated baseline ADA activities in skin and plasma identified to be lower, and decreased baseline erythrocyte ADA was higher.
Utas et al. [35]	Antioxidant Potential of Propylthiouracil in Patients with Psoriasis (2002)	Malondialdehyde (MDA), the end product of lipid peroxidation, superoxide dismutase (SOD) and glutathione peroxidase (GSH-Px), and antioxidant enzymes were measured in plasma, erythrocytes and skin biopsies of psoriatic patients before and after eight weeks of oral treatment with PTU (300 mg/day) or PTU/thyroxine (25 mg). Increased baseline MDA in all samples was found to be lower, and the increased plasma SOD levels in skin and erythrocytes of the study groups were found to be higher and lower, respectively, in all patients after the treatment.
Gnanaraj et al. [36]	"Downregulation of Involucrin in Psoriatic Lesions Following Therapy with Propylthiouracil, an Anti-thyroid Thioureylene: Immunohistochemistry and Gene Expression Analysis" (2015)	Propylthiouracil (PTU), an anti-thyroid thiourylene, is helpful in chronic plaque psoriasis. A precursor protein, Involucrin, is upregulated in psoriasis. In psoriasis, there is an increased expression of involucrin, which leads to abnormal keratinocyte differentiation and the formation of psoriatic plaques. The therapeutic effect of PTU in psoriasis may be attributable to the downregulation of involucrin.

c) Surgery: The possible role of thyroidectomy and thyroid hormones in altering psoriasis's pathogenesis is demonstrated in a 36-year-old Chinese female with an eight-year history of chronic, generalized plaque psoriasis. There is a marked improvement of the disease in this patient after removing the euthyroid multinodular goiter noted [38]. A 61-year-old woman with a one-month history of widespread psoriasis and eight-year symptomatic adenocarcinoma of the thyroid is presented to the hospital. After excision of the carcinoma, there is a marked improvement in her psoriatic lesions and remained in remission without any dermatologic treatment [39].

No Association or Negative Association

1) Common autoimmune diseases tend to accompany the same patients. The association between autoimmune thyroiditis and psoriasis or psoriatic arthritis (PsA) has been observed in a few studies that have examined with inconsistent results. Vassilatou et al. found that psoriatic patients with or without PsA do not have an increased risk for autoimmune thyroiditis [40].

2) Thyroid receptors are expressed in the skin of humans and regulate epidermal proliferation and differentiation. When used TriAc (3,3',5-triiodo-thyroacetic acid) is a well-known thyroid hormone analog with much reduced cardiac thyrotoxic activity compared with the classical thyroid hormones in plaque psoriasis, it is found that it is no more effective than placebo [41].

So far, ligands of thyroid hormone receptors have not been tested as anti-psoriatic agents, further studies needed to explore newer thyroid hormone analogs in treating psoriatic patients.

3) After treatment in a psoriatic patient, there is a significant improvement in T3 and cortisol, and a decreased level of prolactin is observed. Still, there is no statistically significant difference in TSH, T4 levels observed between cases and controls [42].

4) Limited information is available regarding the human leukocyte antigen (HLA) haplotype connection between psoriasis and Hashimoto's thyroiditis. Neither HLA-C nor HLA-B*27 alleles have been implicated in the development of Hashimoto's thyroiditis [43]; thus, evidence regarding an HLA link for psoriasis with Hashimoto's thyroiditis has not been fully elucidated [44].

5) STAT4 is an important transcription factor that activates gene transcription as a response to cytokines. Recently, the STAT4 gene's influence on autoimmune disease has been widely studying in many different immune-related diseases. STAT4 may be a unique gene that switches on in autoimmune-related thyroid disease in psoriatic patients. However, in their study, Hiz et al. found that the STAT4 rs7574865 polymorphism increases autoimmune-related thyroid disease susceptibility among the general population but not in psoriatic patients [16].

6) Khan et al. assess the association of TPO Ab positivity, TSH, and FT4 with the psoriatic disease in the Rotterdam Study, a large prospective population-based cohort study, and performed a systematic review and meta-analysis of available epidemiologic studies evaluating the relation between AITD/thyroid function and psoriatic disease. Within the Rotterdam Study, they found no link between TPO Ab positivity and psoriatic disease. There was a positive link between TSH and the prevalence of psoriatic disease and between the incident of psoriatic disease and FT4, although not significant [45]. However, only a few studies have significant heterogeneity concerning psoriatic disease definition and a sign of publication bias. Supplementary prospective data is needed to assess the link between AITD and the incidence of psoriatic disease.

In this study, we came across the difference in our ground studies, and we can segregate them into two parts. In the first allotted part of the studies, a positive correlation or resonance between the two diseases has been detected. In contrast, another part of the studies showed no connection between the two.

The other face of the study comes across some experiments that nothing clearly stated, a mere coincidence of parameters in both diseases. It cannot ensure a sure shot connection between psoriasis and thyroid or any form of these two diseases. That means more scope for better evaluation and research to assess the two diseases' association.

Limitations

Despite numerous resources and facilities available to collect the data, some sources were inaccessible because they were on a payment basis. Other articles are open to private members citing confidentiality, and some even researched in local languages that required clear interpretation and language skills.

## Conclusions

The gathered data about the association between these diseases showed mixed results. There is not much information about the geographical and genetic link to these two diseases. The majority of the articles show a positive association, and few articles show no connection. Some studies also showed that psoriasis medication might help thyroid problems, and thyroid treatment may help psoriasis. Suppose researchers can establish a connection between them; in that case, including thyroid evaluation (thyroid antibodies and ultrasound ) in psoriasis care might lead to early prevention and treatment of thyroid disease thereby decreasing the cost and efforts associated with these diseases. If these diseases are connected, then developing a universal drug to treat both diseases is possible. Prescribing propylthiouracil as an alternative drug that has fewer side effects for psoriasis patients could be suggested because existing psoriasis treatment is very toxic and expensive. 

More studies are required to establish a connection between these diseases because these findings have a significant impact on both the clinical and research sides.
